# Risk stratification for early mortality in newly diagnosed acute promyelocytic leukemia: a multicenter, non-selected, retrospective cohort study

**DOI:** 10.3389/fonc.2024.1307315

**Published:** 2024-01-30

**Authors:** Suhyeon Kim, Jiye Jung, Seo-Yeon Ahn, Mihee Kim, So Yeon Jeon, Chang-Hoon Lee, Dae Sik Kim, Se Ryeon Lee, Hwa Jung Sung, Chul Won Choi, Byung-Soo Kim, Hyeoung-Joon Kim, Jae-Yong Kwak, Yong Park, Jae-Sook Ahn, Ho-Young Yhim

**Affiliations:** ^1^ Department of Internal Medicine, Jeonbuk National University Medical School, Research Institute of Clinical Medicine of Jeonbuk National University-Biomedical Research Institute of Jeonbuk National University Hospital, Jeonju, Republic of Korea; ^2^ Department of Internal Medicine, Chonnam National University Medical School, Chonnam National University Hwasun Hospital, Jeollanam-do, Republic of Korea; ^3^ Department of Internal Medicine, Korea University College of Medicine Guro Hospital, Seoul, Republic of Korea; ^4^ Department of Internal Medicine, Korea University College of Medicine Ansan Hospital, Ansan, Republic of Korea; ^5^ Department of Internal Medicine, Korea University College of Medicine Anam Hospital, Seoul, Republic of Korea

**Keywords:** acute promyelocytic leukemia, all-trans retinoic acid, early mortality, prognosis, risk stratification

## Abstract

**Introduction:**

Despite the current effective treatments for acute promyelocytic leukemia (APL), early mortality (EM), defined as death within 30 days of presentation, is a major hurdle to long-term survival.

**Methods:**

We performed a multicenter retrospective study to evaluate the incidence and clinical characteristics of EM in patients with newly diagnosed APL and to develop a risk stratification model to predict EM.

**Results:**

We identified 313 eligible patients diagnosed between 2000 and 2021 from five academic hospitals. The median age was 50 years (range 19-94), and 250 (79.9%) patients were <65 years. Most patients (n=274, 87.5%) received their first dose of all-trans retinoic acid (ATRA) within 24 hours of presentation. EM occurred in 41 patients, with a cumulative incidence of 13.1%. The most common cause of EM was intracranial hemorrhage (n=22, 53.6%), and most EMs (31/41, 75.6%) occurred within the first seven days of APL presentation. In a multivariable analysis, we identified three independent factors predicting EM: age ≥65 years (HR, 2.56), white blood cell count ≥8.0 x 10^9^/L (HR, 3.30), and ATRA administration >24 hours of presentation (HR, 2.95). Based on these factors, patients were stratified into three categories with a significantly increasing risk of EM: 4.1% for low risk (54.3%; no risk factors; HR 1), 18.5% for intermediate risk (34.5%; 1 factor; HR 4.81), and 40.5% for high risk (11.2%; 2-3 factors; HR 13.16).

**Discussion:**

The risk of EM is still not negligible in this era of ATRA-based therapies. Our risk model serves as a clinically useful tool to identify high-risk patients for EM who may be candidates for novel treatments and aggressive supportive strategies.

## 1 Introduction

Acute promyelocytic leukemia (APL), a distinct subtype of acute myeloid leukemia (AML), is characterized by the abnormal proliferation of promyelocytes, the presence of profound coagulopathy, and a balanced reciprocal translocation t(15;17) with *PML-RARA* gene fusion (1, 2). The introduction of all-trans retinoic acid (ATRA) and more recently, arsenic trioxide (ATO) in the management of APL has improved treatment outcomes, accounting for cure rates of at least 80% (3–10). However, treatment failure occurs in a substantial number of patients, particularly within the first 30 days of disease presentation (11–17). This early mortality (EM) in APL might be associated with thrombo-hemorrhagic complications such as intracranial hemorrhage (ICH) secondary to a complex coagulopathy at initial presentation (11–17).

Although long-term outcomes have substantially improved with the introduction of ATRA and ATO, previous epidemiological data have suggested that the risk of EM has not improved over time (12, 13), which has led to a growing interest in identifying patients at high risk for EM. Although previous studies have reported clinical risk factors or various risk factor combinations, including age (11, 15, 18), high white blood cell (WBC) count (12, 17–20), hypofibrinogenemia (17), thrombocytopenia (15, 18), prolonged prothrombin time (PT) and activated partial thromboplastin time (aPTT) (21), poor performance status (20), elevated lactate dehydrogenase level (18), and delays in ATRA administration (14, 17), it is still challenging to adequately identify patients with high risk for EM. Österroos et al. (15) recently proposed and externally validated a risk model based on age, WBC count, and platelet count. However, considering the urgent nature of newly diagnosed APL, the clinical use of this model may be hampered by the complexity of calculating risk scores. Moreover, despite the well-established prognostic relevance of early ATRA administration when APL is suspected (14, 17), this has never been investigated as a variable of interest in these risk models (15, 18). Given recent reports that ATRA and ATO-based regimens potentially improve outcomes in patients with APL with high-risk clinical features by reducing the risk of EM (9, 10), it is essential to develop a simple, user-friendly risk stratification model that identifies a specific subgroup of patients at high risk of EM who need aggressive novel preventive strategies.

Therefore, this study aimed to evaluate the incidence of EM and its clinical and laboratory characteristics in patients with newly diagnosed APL and to develop an easy-to-use risk stratification model that integrates clinical risk factors.

## 2 Methods

### 2.1 Study design and patients

We conducted a multicenter, non-selected, retrospective cohort study using data from adult patients consecutively diagnosed with APL at five academic institutions in South Korea between January 2000 and December 2021. Patients were eligible for this study if they were ≥19 years and were newly diagnosed with APL during the study period. The diagnosis of APL required the presence of standard morphological and immunophenotypic features and genetic confirmation (22). Genetic confirmation was established by demonstration of the *PML-RARA* fusion gene using reverse transcriptase polymerase chain reaction, or detection of the t (15, 17) translocation using fluorescence *in situ* hybridization or conventional karyotyping, or both. Patients were excluded if *PML-RARA* fusion or t (15, 17) chromosomal translocation status was uncertain. Patients with incomplete early follow-up data (no survival data within 30 days of disease presentation) were also excluded.

We collected data at each institution using study-specific case report forms after reviewing the medical records, which included clinical and laboratory data at diagnosis, such as age, sex, date and time of first hospital visit related to APL presentation, date and time of first dose of ATRA administered, complete blood count, PT, aPTT, fibrinogen, and D-dimer levels. Information on treatment response, complications during the induction period, disease progression or relapse, and survival, such as date of death or last follow-up, cause of death, and the first date of occurrence of complications related to EM, were based on the data provided by the participating institutions, with no additional central review for the cause of death and disease progression or relapse. The Sanz risk category was determined using the initial WBC and platelet counts (23). The Institutional Review Board of each participating institution reviewed and approved this study and waived the need for written informed consent because the study was retrospective and involved no more than minimal risk to the enrolled patients.

### 2.2 Treatment and supportive care

Patients were admitted to each hospital during induction therapy until they recovered from cytopenia, coagulopathy, and other APL- or therapy-related complications. Most patients were treated with a modified AIDA protocol as induction therapy after the confirmation of APL (3). However, if APL was clinically suspected, ATRA was administered before diagnostic confirmation at the discretion of treating physician. Patients received a conventional dose of ATRA (45 mg/m^2^ orally daily in two divided doses) until complete remission or for a maximum of 60 days, and four doses of intravenous idarubicin (12 mg/m^2^ on days 2, 4, 6, and 8) as induction therapy. Since 2007, intravenous ATO has been used to treat APL on a self-payment basis. Patients were treated with a combination of intravenous ATO at a dose of 0.15 mg/kg and oral ATRA 45 mg/m^2^ daily until complete remission or for a maximum of 60 days after the discussion with their treating physicians.

As this was a retrospective study of real-world practice, the prevention and management of coagulopathy, hyperleukocytosis, and other APL- or therapy-related complications were heterogeneous and usually depended on the policies of each institution. In general, however, to treat coagulopathy during the induction period, platelet and fresh frozen plasma with or without cryoprecipitate were transfused to maintain platelet count ≥30 × 10^9^/L and normalize PT and aPTT with fibrinogen level ≥150 mg/dL until the resolution of significant coagulopathy. Packed red cells were given to maintain hemoglobin level ≥8 g/dL. Prophylactic dexamethasone (4 to 8 mg intravenously every 12 hours) was administered to prevent APL differentiation syndrome for the initial 5 to 10 days of ATRA therapy in patients with an initial WBC count of ≥10 × 10^9^/L and no serious concomitant infections. If symptoms or signs of suspected APL differentiation syndrome developed, ATRA was temporarily discontinued, and dexamethasone (10 mg intravenously twice daily) was administered until the resolution of this adverse event.

Consolidation therapy was primarily based on a modified AIDA protocol during the study period, which consisted of 3 consecutive chemotherapy courses without ATRA (3). However, after 2013, a risk-adapted consolidation strategy according to the AIDA-2000 protocol integrating ATRA for 15 days during each consolidation was used as per the policy of each institution (6). Maintenance therapy with low-dose chemotherapy and ATRA was administered for 2 years (3, 6).

### 2.3 Statistical analysis

The primary endpoint was the cumulative EM rate, which was calculated from the date of the hospital visit related to APL presentation to the date of death within the first 30 days. Patients who survived for ≥30 days were censored on the 30th day. The secondary endpoints included overall survival (OS), which was measured from the date of hospital visit related to APL presentation to the date of death from any cause or last follow-up, and post-30-day OS, which was assessed in patients who survived for ≥30 days and was defined as the interval between 31st day of hospital visit and death from any cause or last follow-up. The Kaplan-Meier method was used to estimate the cumulative rates of EM, OS, and post-30-day OS, and the differences between groups were compared using the log-rank test. To develop a risk stratification model, we performed univariable analysis to assess the association between the cumulative rate of EM and baseline clinical variables. Baseline variables with *P*<0.05 in the univariable analysis were included in the multivariable analysis using the Cox proportional hazards model. Covariates that potentially affected the cumulative rate of EM identified in the multivariable analysis were included to establish a risk model. To simplify the score calculation of the risk model for easy use in clinical practice, we separated continuous variables−age, sex, hemoglobin, WBC and platelet counts, PT, aPTT, fibrinogen level, and period of diagnosis−using receiver operating characteristic (ROC) analysis into two groups based on the best cutoffs for predicting EM. The predictive ability of the established risk model was compared with each risk variable alone or in combination using Harrell’s concordance index (C-index) (24). Descriptive statistics were reported as percentages for categorical variables and median and interquartile range (IQR) for continuous variables. All statistical analyses were performed using R version 4.2.0 (R Foundation for Statistical Computing, Vienna, Austria; http://www.r-project.org).

## 3 Results

### 3.1 Study cohort and baseline patient characteristics

Between January 2000 and December 2021, 323 adult patients with newly diagnosed APL were consecutively enrolled, 10 of whom were ineligible for the study cohort (**Supplementary Figure 1**). Thus, a total of 313 patients were included in this study. The median age was 50 years (range, 19-94), and 250 patients (79.9%) were aged <65 years. The median WBC count was 2.3 × 10^9^/L, and a substantial proportion of patients had abnormalities in coagulation profiles; thrombocytopenia (median platelet count, 27 × 10^9^/L), prolongation of PT (median, 15.9 s) and aPTT (median, 35.6 s), and hypofibrinogenemia (median 152.5 mg/dL). Most patients received the first dose of ATRA within 24 hours of APL presentation; however, this was delayed by more than 24 hours in 39 patients (12.5%). Notably, baseline aPTT and fibrinogen levels were not available for three (1.0%) and 25 (7.9%) patients, respectively. Other baseline laboratory and clinical characteristics are summarized in **Table 1**.

**Table 1:** Clinical characteristics of the study cohort (N=313).

		Total (N=313)
Age, years	Median, range <65 ≥65	50 (19-94) 250 (79.9) 63 (20.1)
Sex	Male Female	154 (49.2) 159 (50.8)
Hemoglobin, g/dL	Median, range <9.4 ≥9.4	8.4 (3.1-17.2) 203 (64.9) 110 (35.1)
White blood cells, ×10^9^/L	Median, range <8.0 ≥8.0	2.3 (0.2-164.8) 234 (74.8) 79 (25.2)
Platelet, ×10^9^/L	Median, range <20 ≥20	27 (1-204) 116 (37.1) 197 (62.9)
PT, sec	Median, range <17.5 ≥17.5	15.9 (8.1-35.9) 230 (73.5) 83 (26.5)
PT, INR	Median, range <1.23 ≥1.23	1.11 (0.83-2.66) 227 (72.5) 86 (27.5)
aPTT, sec*	Median, range <41.5 ≥41.5	35.6 (13.3-67.6) 252 (81.3) 58 (18.7)
aPTT ratio*	Median, range <1.28 ≥1.28	1.18 (0.88-2.13) 202 (64.5) 108 (34.5)
Fibrinogen, mg/dL*	Median, range <90.0 ≥90.0	152.5 (39.6-637.0) 40 (13.9) 248 (86.1)
Sanz category	Low Intermediate High	96 (30.7) 146 (46.6) 71 (22.7)
Timing of first dose of ATRA administered	≤24hours of APL presentation >24hours of APL presentation	274 (87.5) 39 (12.5)
Induction strategies	No ATRA-based ATRA ± anthracycline ATRA + ATO	8 (2.6) 293 (93.6) 11 (3.5)
Period of diagnosis	2000-2011 2012-2021	105 (33.5) 208 (66.5)

*Baseline aPTT and fibrinogen level were not available in 3 (1.0%) and 25 patients (7.9%) at hospital visit related with APL presentation.

PT, prothrombin time; INR, international normalized ratio; aPTT, activated partial thromboplastin time; ATRA, all trans retinoic acid; APL, acute promyelocytic leukemia; ATO, arsenic trioxide.

### 3.2 Cumulative incidence of EM and related characteristics

**Figure 1:**
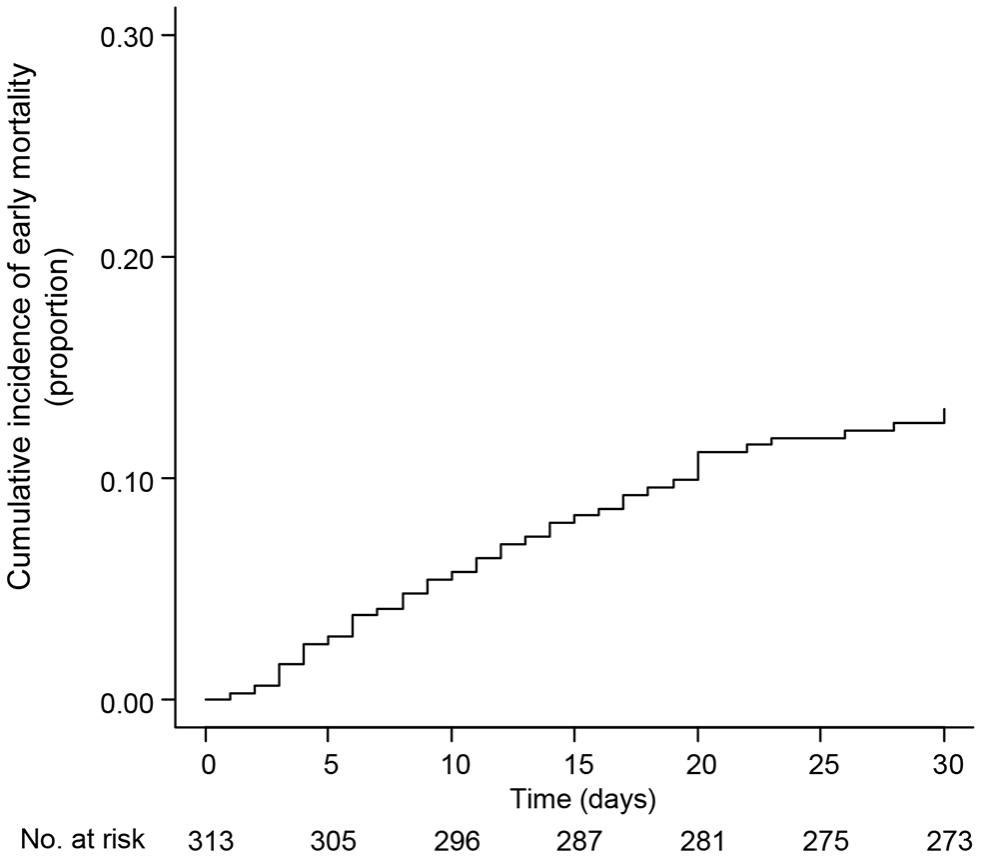
Cumulative incidence of early mortality in the study cohort.

**Figure 2:**
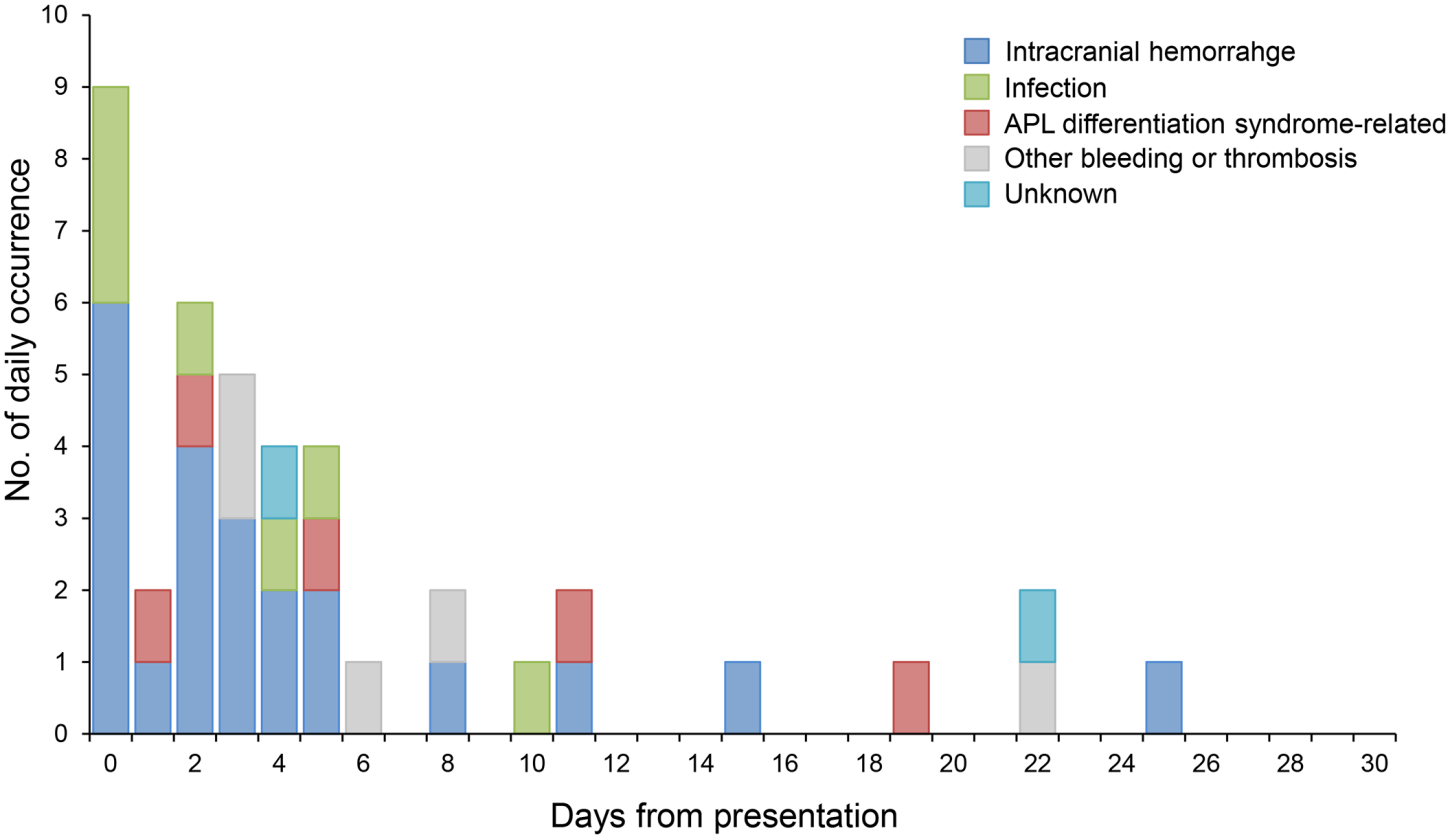
Occurrence of complications related to early mortality. APL, acute promyelocytic leukemia.

During the first 30 days, EM (13.1%) was identified in 41 patients in the study cohort (**Table 2**). The most common cause of EM was ICH, which occurred in 22 patients (53.7%), while other major causes of EM included infection (n=7, 17.1%) and complications associated with APL differentiation syndrome (n=5, 12.2%). The cumulative incidence of EM within 30 days was 13.1% (**Figure 1**). Of the 41 events related to EM, 31 (75.6%) occurred within the first seven days of APL presentation (**Figure 2**). Among the 22 patients with ICH-related EM, 19 (86.4%) occurred within the first 7 days, with a median time to ICH development of 2 days (range, 0−25). Similarly, of the seven infection-related EM, six (85.7%) occurred within the first 7 days.

**Table 2:** Causes of early mortality.

	Total (N=41, %)
Intracranial hemorrhage Infection Complications associated with APL, acute promyelocytic leukemia; differentiation syndrome Other bleeding or thrombosis Unknown	22 (53.7) 7 (17.1) 5 (12.2) 5 (12.2) 2 (4.9)

APL, acute promyelocytic leukemia.

### 3.3 Identification of prognostic factors and risk stratification model development

**Figure 3:**
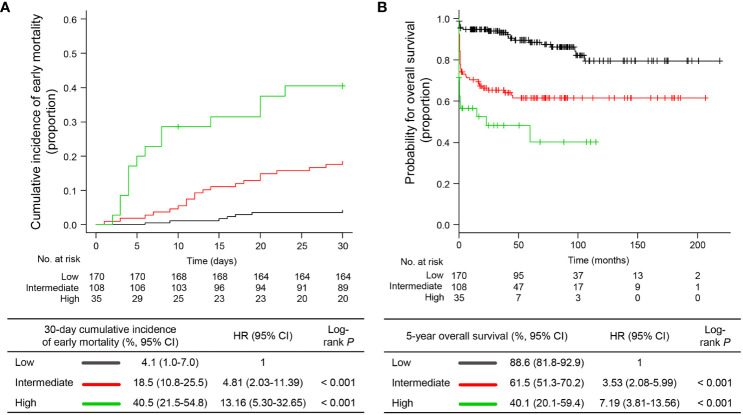
Early mortality and overall survival according to the proposed risk stratification model. **(A)** cumulative incidence of early mortality and **(B)** overall survival. HR, hazard ratio; CI, confidence interval.

To identify the prognostic factors associated with EM, we performed univariable and multivariable analyses based on clinical variables at APL presentation. Continuous variables were divided into two groups using the best cut-offs from the ROC analysis to predict EM (**Supplementary Table 1**). In univariable analysis including age, sex, individual blood cell counts, PT, aPTT ratio, fibrinogen level, Sanz risk category, diagnosis period, and timing of first dose ATRA administration, age ≥65 years (*P*<0.001), WBC ≥8.0 x 10^9^/L (*P*<0.001), PT ≥17.5 sec (*P*=0.001), aPTT ratio ≥1.28 (*P*=0.003), intermediate or high risk by Sanz category (*P*=0.001), and first dose ATRA administration >24 hours after APL presentation (*P*=0.001) were significantly associated with a higher risk of developing EM (**Supplementary Table 2**). By using multivariable Cox proportional hazard analysis including significant variables in the univariable analysis, we identified that age ≥65 years (hazard ratio [HR] 2.56, 95% confidence interval [CI] 1.33−4.91), WBC ≥8.0 x 10^9^/L (HR 3.30, 95% CI 1.76−6.16), and first dose ATRA administration >24 hours after APL presentation (HR 2.95, 95% CI 1.39−6.28) were independent factors for EM (**Table 3**), and these three variables were included in the final model. Each variable independently associated with the risk of EM was scored as one if present or zero if not present. By combining the scores (range, 0−3) for these variables, patients were stratified into three risk groups for EM: low risk (score 0; N=170 [54.3%]), intermediate risk (score 1; N=108 [34.5%]), and high risk (score 2−3; N=35 [11.2%]) groups with 30-day EM rates of 4.1% (95% CI, 1.0−7.0), 18.5% (95% CI, 10.8−25.5), and 40.5% (95% CI, 21.5−54.8), respectively (**Figure 3A**). The established risk model was strongly associated with EM (low *vs.* intermediate, HR 4.81, 95% CI 2.03−11.39, *P*<0.001; low *vs.* high, HR 13.16, 95% CI 5.30−32.65, *P*<0.001; **Figure 3A**) and had the highest predictive ability (C-index, 0.7429) among each risk variable alone or in combination (**Supplementary Table 3**).

**Table 3:** Results of multivariable analysis.

Variables		HR (95% CI)	*P*
Early mortality			
Age, years	<65 ≥65	1 2.56 (1.33−4.91)	0.005
White blood cells, × 10^9^/L	<8.0 ≥8.0	1 3.30 (1.76−6.16)	<0.001
Timing of the first dose of ATRA administered	≤24 hours of APL presentation >24 hours of APL presentation	1 2.95 (1.39−6.28)	0.005
Post-30-day overall survival			
Age, years	<65 ≥65	1 3.23 (1.65−6.33)	<0.001
Sex	Female Male	1 2.19 (1.12−4.29)	0.022
Overall survival			
The established risk model (including age, white blood cells, and timing of the first ATRA administered)	Low Intermediate High	1 3.53 (2.08−5.99) 7.19 (3.81−13.56)	<0.001

ATRA, all trans retinoic acid; APL, acute promyelocytic leukemia; HR, hazard ratio; CI, confidence interval.

### 3.4 Long-term survival outcomes

Of the 272 patients without EM, 38 died during treatment or follow-up, 14 of whom died because of treatment-related complications, 13 died due to APL relapse, and 11 died due to causes unrelated to therapy complications or APL relapse (i.e., five secondary cancers, four ischemic stroke or acute coronary syndrome, and two infections). With a median follow-up of 71.1 months (IQR, 33.3−105.2) and 74.9 months (IQR, 36.7−111.4), the 5-year post-30-day OS and OS were 85.3% (95% CI, 79.8−89.4) and 74.1% (95% CI, 68.4−78.9), respectively. The established risk model was significantly associated with the post-30-day OS (*P*=0.006) and OS (*P*=0.010; **Figure 3B**) in univariable analyses (**Supplemental Table 2**). However, in the multivariable analyses, the risk model was the only significant factor for OS (low *vs.* intermediate, HR 3.53, 95% CI 2.08−5.99; low *vs.* high, HR 7.19, 95% CI 3.81−13.56). However, for post-30-day OS, age ≥65 years (HR 3.23, 95% CI, 1.65−6.33) and male sex (HR 2.19, 95% CI 1.12−4.29) were independent factors (**Table 3**). This is in part because our model specifically reflected the risk of EM. Taken together, we established a risk stratification model including age, WBC count, and timing of ATRA administration to predict the risk of EM in patients with newly diagnosed APL. Our model has better predictive ability than risk variables alone or in combination and identifies a distinctive subgroup of patients with a high risk of death related to expected complications of APL during the early induction period.

## 4 Discussion

In this multicenter retrospective analysis of 313 consecutive patients, the majority of whom received early ATRA administration before the diagnostic confirmation of suspected APL, we observed an EM rate of 13.1%, which was comparable with previous real-world reports (11–17). The main causes of EM and its major occurrence period were also similar to those in previous reports (11–17, 20), with ICH occurring within the first seven days of APL presentation being the most common cause of death. We identified clinical variables related to an increased risk of EM: age ≥65 years, initial WBC ≥8.0 x 10^9^/L, and delayed ATRA administration >24 hours after disease presentation. Based on these observations, we developed a simple, user-friendly risk stratification model that identified specific subgroups of patients with a substantial risk of EM. Moreover, the risk stratification model was an independent prognostic factor for OS. These findings indicate that our risk model may serve not only as an indicator of EM but also as a relevant surrogate for long-term outcomes in APL; thus, novel treatment strategies that reduce the risk of EM need to be explored in this higher-risk patient subgroup.

ATRA alleviates the coagulopathy associated with APL by differentiating leukemic cells into terminal neutrophils (25, 26). Early administration of ATRA in patients with suspected APL is crucial for reducing the risk of coagulopathy-related EM and for long-term survival (1, 27). In the present study, patients with an early ATRA treatment ≤24 hours of presentation had better outcomes with low rates of EM and improved long-term survival than those with a delayed ATRA administration, consistent with previous reports (14, 17). The reduced risk of EM in patients with early ATRA administration may not only be related to the pharmacological effect of ATRA but also be attributed to an immediate institution of aggressive supportive care based on the clinical suspicion of APL. These findings emphasize the importance of a high awareness of this disease among physicians. In this study, 87.5% of the patients received early ATRA treatment within 24 hours of presentation, suggesting a substantially high awareness of this disease. However, despite such a high level of clinical suspicion for APL, this potentially fatal disease was still not suspected in approximately 10-15% of patients during the initial presentation, based on the missing data for blood coagulation tests in the study cohort. Therefore, maintaining a high awareness among physicians regarding early suspicion for the potential diagnosis of APL, prompt ATRA administration, and aggressive supportive measures to counteract coagulopathy in patients presenting with hemorrhagic symptoms and laboratory evidence of coagulopathy with characteristic morphology of leukemic cells on a blood smear would be of paramount importance in reducing the risk of EM in this highly curable disease.

However, despite early ATRA-based therapy and aggressive blood product replacement strategies in our study cohort, the established risk stratification model can identify subgroups of patients with a substantially increased risk of EM. In this study, the 30-day EM rates among patients in the intermediate and high-risk subgroups were 18.5% and 40.5%; 80% of patients were 65 years or older and 60% had a WBC count ≥8.0 x 10^9^/L in the high-risk subgroup, indicating considerable room for improvement. In this aspect, there has been great interest in combining ATRA with ATO for newly diagnosed APL. In a pivotal prospective randomized trial comparing ATRA and ATO to ATRA and idarubicin treatments (8), the ATRA and ATO combination was at least equivalent and possibly superior to the ATRA-idarubicin combination because of the reduced risk of hematological adverse events and subsequent lower mortality of the ATRA-ATO combination. However, this trial only included patients with age<71 years and WBC ≤10.0 x 10^9^/L, which limited to provide the role of ATO-based regimens in patients with APL with high-risk clinical features. In another randomized trial, the AML17 trial (9), investigators compared the ATRA-ATO combination with ATRA and idarubicin, irrespective of age or WBC count, and demonstrated that the ATRA and ATO combination was feasible in patients with or without high-risk clinical features and showed less 30-day and 60-day mortality with lower occurrences of hemorrhagic death, although statistical significance was not observed. Other ATRA and ATO-based approaches, including an adjusted dose of idarubicin for age, also reported promising results in the high-risk group, showing better survival outcomes in patients treated with ATO-containing therapy translated from non-significant reduction in EM and absence of death in remission than those without ATO treatment (10). Moreover, as specific genetic abnormalities in non-APL AML can predict a poor response to certain chemotherapeutic agents (28), additional molecular aberrations in APL may be associated with primary ATRA resistance and consequent potential risk of EM (29, 30). The *FLT3-ITD* mutation is commonly present in APL (31), associated with high WBC count, a well-known high risk clinical feature for EM. In the recent experimental study reported by Esnault and colleagues (32), *FLT3-ITD* mutation was found to inhibit the degradation of PML-RARA protein, potentially contributing to blunting the therapeutic effects of ATRA and leading to ATRA resistance. Interestingly, the researchers demonstrated that such resistance could be overcome by ATRA and ATO combination. These findings suggest a potential therapeutic role for ATRA and ATO-based combination treatment in improving outcomes of patients with high-risk clinical or molecular features by reducing the risk of EM. Furthermore, there are encouraging data regarding the role of recombinant soluble thrombomodulin in coagulopathy associated with hematological malignancies or infection (33, 34). Thus, prospective trials incorporating ATO and other novel agents to counteract coagulopathy should be preferentially explored in these subgroups with high-risk clinical features. Our risk stratification model may serve to identify patients who may benefit the most from these novel approaches.

This study had several limitations that require careful consideration. First, this was a retrospective study, and there may have been unrecognized selection bias. In addition, the absence of external validation may limit the generalizability of our findings. Second, we analyzed continuous clinical variables as dichotomized factors using the best cutoffs from the ROC analysis to develop a simple and easy-to-use clinical risk model. The relatively small number of patients included in this study might have affected the selection of alternative cutoff values that differed from previously suggested values. In particular, WBC count ≥10.0 x 10^9^/L was the commonly used cutoff associated with poor outcomes in APL (6, 8–10, 23). However, we adopted a WBC ≥8.0 x 10^9^/L cutoff because this was more predictive for EM in our study cohort. Furthermore, recent data suggest that EM is already increasing with WBC counts below or within the normal range (12, 15). Thus, further studies with larger populations are needed to define the optimal cut-off WBC count for predicting the risk of EM. Third, the present risk model was developed by using baseline clinical variables, but other biologic features that might affect the outcomes were not considered. The data on additional molecular aberrations (29) and other molecular biomarkers that have shown the prognostic influence in APL were not consistently available in this study. Thus, further study are needed to evaluate the impact of these biological features on the risk of EM. Fourth, we included over 20 years, and the results may have been affected by improvements in supportive care over the study period. In particular, the availability of ATO in salvage setting during the study period may have affected OS (35). However, we could not capture temporal improvements in the EM and OS rates in this study cohort, suggesting that the impact of evolving treatments on survival outcomes was minimal. Fifth, because this is a multicenter retrospective study with long follow-up period, data on the amount and timing of blood product transfusion were not available to analyze the impact of transfusion on the risk of EM. The information on the management of APL-related comorbidities was not available for the similar reason.

In conclusion, this multicenter cohort study shows a substantial risk of EM even in the ATRA-based therapy era and establishes a risk stratification model using age, WBC count, and timing of ATRA administration for predicting EM in patients with APL. The risk model identifies approximately one-half of the patients with a low risk of EM. These patients can be treated with the current ATRA-based combination treatments (i.e., ATRA-idarubicin or ATRA-ATO combinations) with appropriate supportive care based on the availability of ATO in each institution. In addition to early ATRA treatment, patients in the intermediate- or high-risk groups, especially those with additional molecular abnormalities, should be targeted for studies incorporating ATO and other novel therapeutics, accompanied by aggressive supportive measures to reduce the risk of coagulopathy-related EM during the early induction period. Therefore, our model may be used to design future studies investigating risk-stratified treatment strategies for APL. Our model and observed findings warrant validation in a large external cohort.

## Data availability statement

The raw data supporting the conclusions of this article will be made available by the authors, without undue reservation.

## Ethics statement

The studies involving humans were approved by The Institutional Review Board. The studies were conducted in accordance with the local legislation and institutional requirements. The ethics committee/institutional review board waived the requirement of written informed consent for participation from the participants or the participants’ legal guardians/next of kin because the study was retrospective and involved no more than minimal risk to the enrolled patients.

## Author contributions

SK: Conceptualization, Data curation, Formal analysis, Investigation, Validation, Visualization, Writing – original draft, Writing – review & editing. JJ: Conceptualization, Data curation, Formal analysis, Investigation, Validation, Visualization, Writing – original draft, Writing – review & editing. S-YA: Conceptualization, Data curation, Formal analysis, Investigation, Validation, Visualization, Writing – original draft, Writing – review & editing. MK: Data curation, Validation. SJ: Data curation, Validation. C-HL: Data curation, Validation. DK: Data curation, Validation. SL: Data curation, Validation. HS: Data curation, Validation. CC: Data curation, Validation. B-SK: Data curation, Validation. H-JK: Data curation, Validation. J-YK: Data curation, Validation. YP: Conceptualization, Data curation, Formal analysis, Investigation, Methodology, Project administration, Supervision, Validation, Visualization, Writing – original draft, Writing – review & editing. J-SA: Conceptualization, Data curation, Formal analysis, Investigation, Methodology, Project administration, Supervision, Validation, Visualization, Writing – original draft, Writing – review & editing. HY-Y: Conceptualization, Data curation, Formal analysis, Investigation, Methodology, Project administration, Supervision, Validation, Visualization, Writing – original draft, Writing – review & editing.
