# Supplementary material for: Risk stratification for early mortality in newly diagnosed acute promyelocytic leukemia: a multicenter, non-selected, retrospective cohort study
Source: Front Oncol. 2024 Jan 30;14:1307315. doi: 10.3389/fonc.2024.1307315 (PMC10861669; doi:10.3389/fonc.2024.1307315)
Supplement: Supplementary file 1 [file DataSheet_1.doc]

**Supplementary Information**

Suhyeon Kim, Jiye Jung, and Seo-Yeon Ahn et al.

**Table of Content**

1. Supplemental Table 1. Determination of best cutoff for continuous variables (Page 2)
2. Supplemental Table 2. Univariable analysis for early mortality(Page 3)
3. Supplemental Table 3. Comparison of predictive ability of the established risk model with each risk variable alone or in combination (Page 4)
4. Supplemental Figure 1. Study cohort (Page 6)

**Supplemental Table 1. Determination of best cutoffs for continuous variables**

| Continuous variables | cutoff | Sensitivity (%) | Specificity (%) | AUC |
| --- | --- | --- | --- | --- |
| Age, years | 65 | 43.9 | 83.5 | 0.671 |
| Hemoglobin, g/dL | 9.4 | 43.9 | 66.2 | 0.538 |
| White blood cells, × 10^9^/L | 8.0 | 48.8 | 79.9 | 0.679 |
| Platelet, , × 10^9^/L | 20 | 65.9 | 37.5 | 0.490 |
| PT, sec | 17.5 | 48.8 | 76.8 | 0.621 |
| PT, INR | 1.23 | 39.0 | 82.0 | 0.610 |
| aPTT, sec | 41.5 | 31.7 | 83.3 | 0.558 |
| aPTT ratio | 1.28 | 58.5 | 68.8 | 0.574 |
| Fibrinogen, mg/dL | 90.0 | 76.3 | 20.0 | 0.579 |
| Period of diagnosis, calendar year | 2012 | 51.2 | 50.0 | 0.435 |

Given that higher AUC value indicates better discriminative performance, we conduct the further analysis for PT and aPTT using PT (sec) and aPTT ratio in this study.

Abbreviations: PT, prothrombin time; INR, international normalized ratio; aPTT, activated partial thromboplastin time; AUC, area under curve.

**Supplemental Table 2: Univariable analysis for early mortality, post-30-day overall survival, and overall survival.**

| Variables | Patients (n=313, %) | Cumulative incidence of early mortality within 30 days | | Post-30-day overall survival | | Overall survival | |
| --- | --- | --- | --- | --- | --- | --- | --- |
|  |  | HR (95% CI) | P value | HR (95% CI) | P value | HR (95% CI) | P value |
| Age, years  <65  ≥65 | 250 (79.9)  63 (20.1) | 1  3.49 (1.88-6.48) | <0.001 | 1  3.38 (1.73-6.63) | <0.001 | 1  3.44 (2.18-5.42) | <0.001 |
| Sex  Female  Male | 159 (50.8)  154 (49.2) | 1  1.21 (0.65-2.23) | 0.541 | 1  2.30 (1.17-4.51) | 0.015 | 1  1.64 (1.05-2.58) | 0.030 |
| Hemoglobin, g/dL  ≥9.4  <9.4 | 110 (35.1)  203 (64.9) | 1  1.47 (0.79-2.73) | 0.215 | 1.00 (0.50-1.99) | 0.990 | 0.80 (0.51-1.27) | 0.359 |
| White blood cells, ×10^9^/L  <8.0  ≥8.0 | 234 (74.8)  79 (25.2) | 1  3.15 (1.70-5.81) | <0.001 | 1.38 (0.67-2.85) | 0.378 | 2.18 (1.35-3.44) | 0.001 |
| Platelet, ×10^9^/L  ≥20  <20 | 197 (62.9)  116 (37.1) | 1  0.86 (1.88-4.12) | 0.655 | 1  1.16 (0.60-2.22) | 0.650 | 1  1.00 (0.63-1.58) | 0.997 |
| PT, sec  <17.5  ≥17.5 | 230 (73.5)  83 (26.5) | 1  2.88 (1.56-5.31) | 0.001 | 1  1.53 (0.77-3.03) | 0.223 | 1  2.15 (1.37-3.37) | 0.001 |
| aPTT ratio  <1.28  ≥1.28 | 202 (64.5)  108 (34.5) | 1  2.77 (1.49-5.16) | 0.003 | 1  1.70 (0.87-3.19) | 0.157 | 1  1.84 (1.08-3.01) | 0.031 |
| Fibrinogen, mg/dL  ≥90.0  <90.0 | 248 (86.1)  40 (13.9) | 1  1.40 (0.49-3.95) | 0.520 | 1  3.10 (0.74-12.91) | 0.120 | 1  1.97 (0.85-4.54) | 0.111 |
| Sanz category  Low  Intermediate  High | 96 (30.7)  146 (46.6)  71 (22.7) | 1  1.91 (0.75-4.86)  4.65 (1.84-11.73) | 0.001 | 1  0.88 (0.42-1.81)  1.11 (0.46-2.68) | 0.855 | 1  1.20 (0.68-2.11)  2.27 (1.25-4.11) | 0.011 |
| Timing of the first dose of ATRA administered  ≤24 hours of APL presentation  >24 hours of APL presentation | 274 (87.5)  39 (12.5) | 1  3.03 (1.51-6.05) | 0.001 | 1  1.77 (0.74-4.25) | 0.198 | 1  2.41 (1.41-4.14) | 0.001 |
| Consolidation therapy  with ATRA  without ATRA | 251 (100.0)  198 (78.9)  53 (21.1) | − | − | 1  1.25 (0.54-2.88) | 0.592 | 1  1.25 (0.54-2.88) | 0.592 |
| The established risk model  Low  Intermediate  High | 170 (54.3)  108 (34.5)  35 (11.2) | − | − | 1  2.91 (1.47-5.77)  3.13 (1.02-9.55) | 0.006 | 1  3.53 (2.08-5.99)  7.19 (3.81-13.56) | 0.010 |

Abbreviations: HR, hazard ratio; CI, confidence interval; PT, prothrombin time; aPTT, activated partial thromboplastin time; ATRA, all-trans retinoic acid; APL, acute promyelocytic leukemia.

Supplemental Table 3: Comparison of predictive ability of the established risk model with each risk variable alone or in combination

| **Variables** | **C-index** |
| --- | --- |
| Age alone  WBC count alone  Timing of first dose of ATRA administration alone | 0.6289  0.6311  0.5832 |
| Age and WBC count  Age and timing of first dose of ATRA administration  WBC count and timing of first dose of ATRA administration | 0.7179  0.6891  0.6665 |
| The established risk model (including age, WBC count, and timing of first dose of ATRA administration) | 0.7429 |

The C-index provides a measure of discriminative ability of each model. The higher values of C-index denotes the better performance of the model.

Abbreviations: C-index, concordance index; WBC, white blood cells; ATRA, all trans retinoic acid.

**Supplemental Figure 1. Study cohort.**


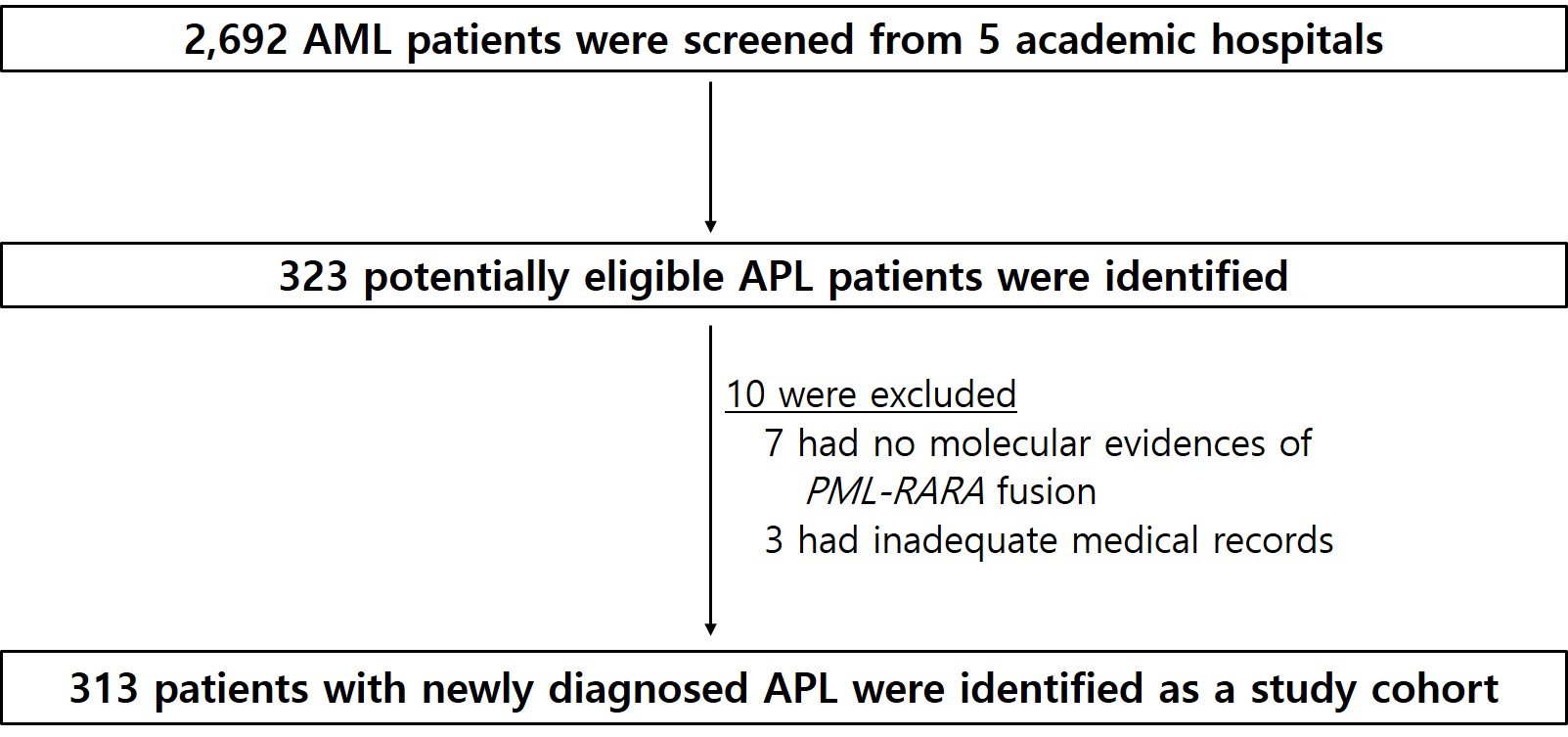


Abbreviations: AML, acute myeloid leukemia; APL, acute promyelocytic leukemia.
